# Lipidomic analysis of cancer cells cultivated at acidic pH reveals phospholipid fatty acids remodelling associated with transcriptional reprogramming

**DOI:** 10.1080/14756366.2020.1748025

**Published:** 2020-04-20

**Authors:** Lorena Urbanelli, Sandra Buratta, Mariantonia Logozzi, Nico Mitro, Krizia Sagini, Rossella Di Raimo, Donatella Caruso, Stefano Fais, Carla Emiliani

**Affiliations:** a Department of Chemistry, Biology and Biotechnology, University of Perugia, Perugia, Italy; b Department of Oncology and Molecular Medicine, National Institute of Health, Rome, Italy; c Department of Pharmacological and Biomolecular Sciences, University of Milan, Milan, Italy; d CEMIN-Center of Excellence for Innovative Nanostructured Material, University of Perugia, Perugia, Italy

**Keywords:** Phospholipid remodelling, desaturases, elongases, tumour pH, tumour microenvironment

## Abstract

Cancer cells need to modulate the biosynthesis of membrane lipids and fatty acids to adapt themselves to an accelerated rate of cell division and survive into an extracellular environment characterised by a low pH. To gain insight this crucial survival process, we investigated the lipid composition of Mel 501 melanoma cells cultured at either physiological or acidic pH and observed the remodelling of phospholipids towards longer and more unsaturated acyl chains at low pH. This modification was related to changes in gene expression profile, as we observed an up-regulation of genes involved in acyl chain desaturation, elongation and transfer to phospholipids. PC3 prostate and MCF7 breast cancer cells adapted at acidic pH also demonstrated phospholipid fatty acid remodelling related to gene expression changes. Overall findings clearly indicate that low extracellular pH impresses a specific lipid signature to cells, associated with transcriptional reprogramming.

## Introduction

It is widely recognised that tumour cells need to adapt their metabolism, in order to survive and proliferate in an altered microenvironment. This adaptation requires not only the reprogramming of energy and nucleotide metabolism to support DNA replication, but also the biosynthesis of fatty acids and membrane lipids, which are necessary to maintain cell identity and support accelerated cell division^1^.

Lipids have been demonstrated to be involved in carcinogenic processes^2^. In membranes, they form specialised domains that are important for protein activation and signal transduction. From a structural point of view, changes in lipid composition may affect membrane fluidity and protein dynamics, altering signalling pathways which are known to be dysregulated in cancer and involved in uncontrolled growth, malignant transformation and metastasis^3^. In addition, membrane lipids have a direct role in signalling as lipid mediators in pathways which have been shown to be altered in tumour development^4^. Beyond signal transduction, alteration of the lipid composition may additionally contribute to carcinogenesis. In fact, the enrichment in cholesterol and saturated fatty acids has been correlated to an increased rigidity of tumour cell membranes, which protects cells from oxidative damage and inhibits the uptake of chemotherapeutic drugs^5^. On the other hand, a low content of cholesterol may confer to membranes an increased deformability, rendering them more prone to enter blood vessels and increasing their metastatic potential^6^. Lipid metabolism alterations have been also increasingly recognised as key events in cancer metastasis and recent genome-wide screens have demonstrated that metastatic cells express high levels of lipid metabolism or lipid transport genes^7^^,^^8^. In fact, tumour cells need a balancing of lipid anabolic and catabolic reactions to meet the increasing need of nutrients and energy. The capacity to reprogramme lipid metabolism confers a selective advantage to metastatic cells.

A well-recognised and common metabolic phenotype observed in most types of solid tumours is the elevated rate of fermentative glycolysis, i.e. the non-oxidative conversion of glucose to lactic acid. While this can be induced as an adaptive response to poor oxygenation (the “Pasteur Effect”), a remarkable century old observation is that this glycolytic phenotype can be hardwired, and thus cancers ferment glucose even in the presence of adequate oxygen (the “Warburg Effect”). Although the mechanism and drivers of glycolytic switch are still debated^9^^,^^10^, it is an unequivocal fact that tumours are characterised by the accumulation of H^+^ in their microenvironment leading to its acidification, with values as low as pH 6.5^11–14^. This acidic microenvironment strongly influences cancer progression and evolution^12^. Although it is initiated early in carcinogenesis, it is retained as cancers become locally invasive, a process known as “niche engineering”. As acidity is evident in early cancers, it could possibly contribute to intratumoral genetic heterogeneity or even to a sort of microevolutionary process^12^^,^^15^.

Cells must adapt their metabolic repertoire to low extracellular pH to maintain membrane integrity. Low extracellular pH has been shown to influence lipid peroxidation processes^16^ and additional changes in lipid composition could in turn affect transmembrane protein dynamics, including the activity of proteins involved in maintaining the gradient of pH between the intracellular and extracellular environment and between the cytoplasm and subcellular organelles, i.e. as proton pumps and ion transporters^17^. However, it is not known whether the low pH conditions of tumour microenvironment may change the lipid composition of tumour cells as well. Previous studies investigated melanoma cells lipid composition in stress conditions. In response to temperature increase cells undergo membrane lipid remodelling characterised by increase in saturated lipid species and phosphatidylinositol (PI)^18^, whereas UVA exposure reduced the content of phosphatidylcholine (PC) and incremented that of PI^19^. Consistent findings, i.e. a progressive increase of specific species of PI in association with an increasing metastatic potential of melanoma cells, has been also recently reported^20^.

In this study, we investigated in detail the lipid composition of human melanoma cells (Mel501), with respect to the same cells adapted to grow at acidic conditions^21^^,^^22^, an environmental perturbation that profoundly and stably change melanoma cell morphology and its invasive behavior^23–25^. We demonstrated that the lipid signature of melanoma cells changes in a peculiar manner when cells are adapted to growth at pH 6.5. These changes are accompanied by a specific transcriptional reprogramming of genes involved in fatty acids and phospholipid remodelling. Further analysis confirmed gene expression modification and fatty acids remodelling in PC3 prostate and MCF7 breast cancer cell lines adapted at low pH.

## Materials and methods

### Cell cultures

Human melanoma cell line Mel501 was adapted to grow at acidic conditions as described in previous studies^21^^,^^22^^,^^26^. The same protocol was adopted for MCF7 breast cancer cell line and PC3 prostate cancer cell lines. Cells were negative for Mycoplasma contamination (Venor^®^GeM, Minerva Biolabs, Germany). Briefly, cells were cultured in buffered medium RPMI 1640 (pH 7.4) (Mel501, MCF7, PC3) and in RPMI 1640 unbuffered medium without sodium bicarbonate, at pH 6.5 (Mel501ac, MCF7ac, PC3ac), supplemented with 10% Foetal Calf Serum and 1% Penicillin/Streptomycin. The acid medium (pH 6.5), and intermediate pH values 7.0 and 6.75 were obtained adding 1 M HCl solution to RPMI medium. The pH was measured using a pH 123 Microprocessor pH Metre (Hanna Instruments, Milan, Italy). To grow at pH 6.5, cells were adapted passing to 7.2, 6.9, 6.75 and to 6.5, in a time ranging from three to four weeks depending on cell type, allowing the cells to not be exposed to short-term pH stress.

### Cell viability assay

Cells in buffered and acid medium were seeded in 12 well-plates. After 24 h, cells were centrifuged and stained with 0.05% Trypan Blue (Lonza) for 10 min. Then cell death was determined with BD FACSCalibur flow cytometer and analysed with CellQuestPro (Becton Dickinson Systems). Alternatively, cell viability was measured by in Countess Automated Cell Counter using Trypan Blue Stain (0.4%) (ThermoFischer Scientific).

### Invasion analysis

Mel501 and Mel501ac cells (3 × 10^6^) were seeded in Cell Culture Dishes and incubated overnight in humidified incubator. Cell monolayers were scraped with a p1000 pipet tip and debris were washed with PBS. Fresh medium was added, and dishes placed in culture incubator at 37 °C. After 18 h, dishes were observed under a phase-contrast microscope and images acquired.

### Immunocytochemistry

Mel501 and Mel501ac (3 × 10^4^ cells per chamber) were attached to sterile glass chamber slides (LabTek, Naperville, IL) by overnight incubation with 150 µl of RPMI 1640 per well in humidified 5% CO_2_ atmosphere. Then, cells were fixed in cold 70% methanol (10 min at 4 °C) and stained with mouse monoclonal anti-LAMP-2 (Abcam) using the peroxidase anti-peroxidase method in single staining.

### Cells preparation for lipidomic profile

Cells were trypsinized, washed twice with PBS at 4° C and centrifuged again. About 7 × 10^6^ of either Mel501 cultivated at pH 7.4 or Mel501 cultivated in acidic conditions at pH 6.5 were pelleted and stored at −80° C prior to analysis. Total cellular lipids were extracted from 6 pellets of Mel501 and 4 pellets of Mel501ac and protein concentration determined in each sample to normalise lipid content. Extracts were dried under nitrogen and resuspended in methanol prior to be submitted for analyses.

### Lipid profile by liquid chromatography-tandem mass spectrometry (LC-MS/MS)

Internal standard (IS) for cholesterol (5α-cholestane) and fatty acids ([U13C18]-linoleic acid) were added to samples and lipids extraction was performed with the Folch method (chloroform/methanol (MeOH) 2:1 v/v). Samples were left o/n at 4 °C. Then, the organic residue was divided in two fractions: one for the analysis of phospholipids and free fatty acids (fraction A, 60% of the total sample), and the other for analysis of total cholesterol and fatty acids (fraction B, 40% of the total sample). Total cholesterol and fatty acids were obtained from samples by acid hydrolysis as previously described^27^. Briefly, fraction B was resuspended in chloroform/MeOH 1:1 v/v. 1 M HCl:MeOH (1:1, v/v) was added to the total lipid extract and shook for 2 h. Then, chloroform:water (1:1 v/v) was added, and the lower organic phase was collected, split, transferred into tubes and dried under nitrogen flow.

For the quantification of the different phospholipid families (fraction A) the MS analysis was performed with a flow injection analysis-tandem mass spectrometry (FIA-MS/MS) method as previously described^28^. The identity of the different phospholipid families was confirmed using pure standards, namely one for each family. Methanolic extracts of cells were analysed by a 3 min run in both positive and negative ion mode with a 268 multiple reaction monitoring (MRM) transition in positive mode and 88 MRM transition in negative mode. An ESI source connected with an API 4000 triple quadrupole instrument (AB Sciex, USA) was used. The mobile phase was 0.1% formic acid in MeOH for FIA positive analysis and 5 mM ammonium acetate pH 7 in MeOH for FIA negative. MultiQuant™ software version 3.0.2 was used for data analysis and peak review of chromatograms. Semi-quantitative data were normalised on the protein content of cells.

### Total and free fatty acids quantification

Sample for free (fraction A) and total fatty acids (fraction B) were suspended in MeOH, concentrated and transferred into auto-sampler for LC-MS/MS analyses. The quantification was performed with a selective ion monitoring-tandem mass spectrometry (SIM-MS/MS) method. An ESI source connected with an API 4000 triple quadrupole instrument (AB Sciex, USA) was used. Chromatographic separation was performed using Hypersil GOLD C8 column (100 mm × 3 mm, 3 µm) maintained at 40 °C. The mobile phases were: 10 mM isopropylethylamine/15 mM acetic acid in water: MeOH 97:3 (phase A) and methanol (phase B). The gradient (flow rate 0.5 ml/min) was as follows: T0: 80% A, T20: 99% A, T25; 99% A, T25.1: 80% A, T30: 80% A. The mass spectrometer was operated in negative ion mode. MultiQuant™ software version 3.0.2 was used for data analysis and peak review of chromatograms. Quantitative evaluation of different fatty acids was performed based on standard curves freshly prepared, using a fixed concentration of the IS, and different concentrations of fatty acids analysed. Quantitative data were normalised on the protein content of cells.

### Cholesterol analysis

Total cholesterol quantitative analysis was performed as previously described^41^. Briefly, an aliquot of fraction B was first derivatized with a mixture of bis-trimethylsilyltrifluoroacetamide (BSTFA): pyridine (4:1 v/v) for 30 min at 60 °C, and then injected into a gas chromatograph-mass spectrometer (GC-MS, Varian Saturn 2100). The MS was operated in the electron impact (EI) ionisation mode. GC-MS analysis was performed as follows: 1 μl sample was injected in splitless mode (inlet was kept at 270 °C with the helium flow at 1.0 ml/min) at the initial 180 °C. The oven was first kept at 180 °C for 1 min, ramped at 50 °C/min to 240 °C, then at 5 °C/min to 300 °C for 6 min. The ions used for the quantification of cholesterol were at m/z 368 for cholesterol and m/z 357 for 5α-cholestane, the IS. The selection of ions for selective ion monitoring (SIM) analysis was based on mass spectra of pure standards and the quantification was based on calibration curves freshly prepared using a fixed concentration of the IS, and different concentrations of cholesterol, in a range from 0 to 10 μg/μl. Quantitative data were normalised on the protein content of cells.

### Quantitative PCR

RNA was extracted using Trifast reagent (Euroclone), according to the manufacturer’s instructions. 1 μg of RNA was reverse-transcribed into cDNA using random hexamers and SuperScript II Reverse Transcriptase (Life Technologies, Carlsbad, CA, USA). cDNA was used to determine transcript levels by qRT-PCR in a StepOne RT-PCR machine (Applied Biosystems, USA) using SYBR^®^ Select Master Mix (Life Technologies). Primers used are listed in Supplementary Table 1. GADPH used as endogenous control was amplified with primers 5′-GAG AAG GCT GGG GCT CAT TT (forward) and 5′-AGT GAT GGC ATG GAC TGT GG (reverse). Data were analysed using the ΔΔCt method. ΔCt was calculated subtracting the average Ct value of GADPH as control to the average Ct value of each transcripts for Mel501 and Mel 501ac. ΔΔCt is the difference between the ΔCt for each transcript for Mel501ac and the ΔCt of each transcript for Mel501 as control. The reported fold expression, expressed as RQ (Relative Quantity), was calculated by 2-ΔΔCt. The analysis was repeated three times in triplicate. The mean ± SD of a representative experiment is reported (*p < 0.05).

### Statistical analysis

Statistical comparison was performed using Student’s *t*-test. Differences were considered statistically significant when *p* < 0.05. For lipid analysis, 6 independent experiments for Mel 501 cultivated at pH 7.4 and 4 for Mel501 cultivated at pH 6.5 (Mel 501ac) were carried out.

## Results

### Mel501 viability and behaviour at low pH condition

**Figure 1.**
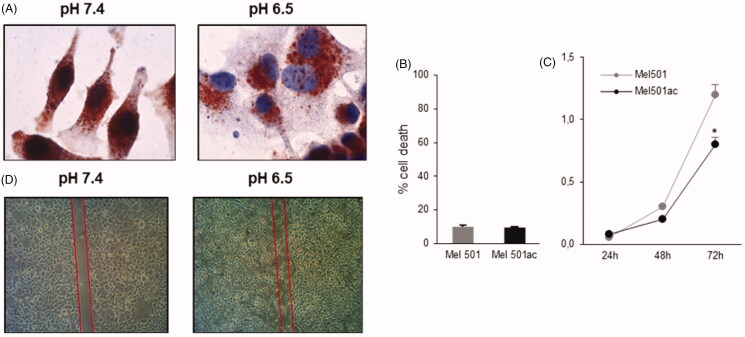
Analysis of Mel501 vitality and growth properties in buffered (Mel501) and in acidic conditions (Mel501ac). (A) Immunocytochemistry assay. Cells were fixed in cold 70% methanol and stained with mouse monoclonal anti-LAMP-2 using the peroxidase anti-peroxidase method in single staining. Images were acquired at 60× original magnification. (B) Cell viability assay. Cells were stained with 0.05% Trypan Blue for 10 min and cell death was determined by flow cytometry. The percentage of cells incorporating Trypan Blue with respect to total cells counted is reported. (C) Growth curve of Mel501 adapted at low pH. The cell number is reported. Mean values were calculated on 3 replicates and mean ± s.d. is indicated for each sample. *p < 0.05. (D) Wound closure assay. Cells were scraped, and debris washed with PBS. After 18 h, dishes were observed under a phase-contrast microscope and images were acquired, 10× original magnification.

Cancer cells, including melanoma cells, change their morphology and show high invasive potential when grown at low pH values^23–25^, comparable to those within a tumour mass^11–14^. In a first set of experiments we confirmed data obtained in previous studies^21^^,^^22^, showing that Mel501 melanoma cells adapted to grow at pH 6.5 displayed different morphological features, as they appeared flattened and enlarged (Figure 1(A)), with a more intense staining for the lysosomal compartment marker Lamp-2, demonstrating the enlargement of intracellular vesicular compartment previously shown^29^. These cells also displayed a slower turnover rate (Figure 1(C)), without any differences in viability (Figure 1(B)). A behaviour consistent with a higher migration potential, with respect to the same cells cultivated at pH 7.4, was observed by wound healing assay (Figure 1(D)), in agreement with previous reports^21–22^.

### Lipid class composition of Mel501 cells cultured in buffered (Mel501) and in acidic conditions (Mel501ac)

**Figure 2.**
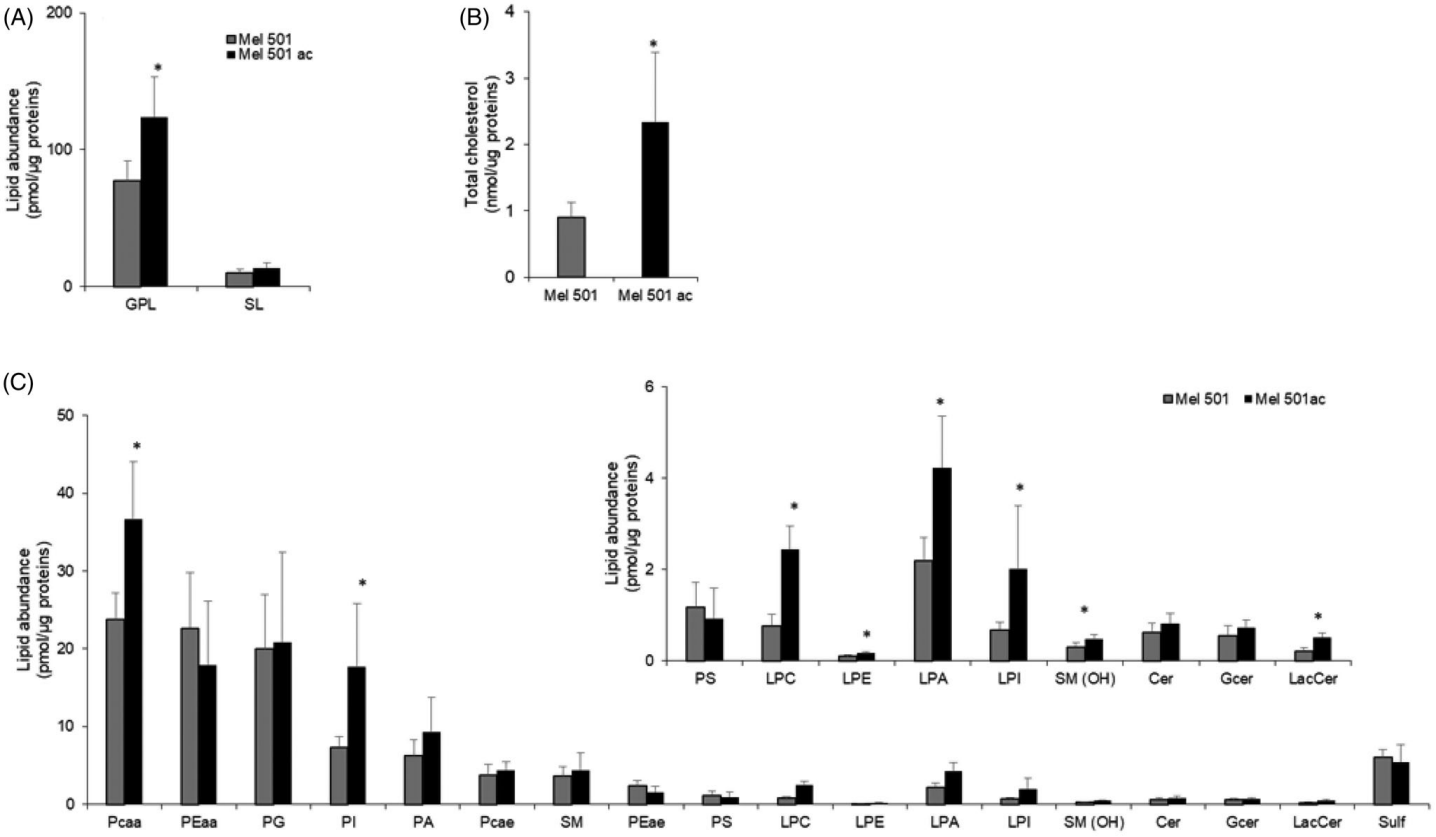
Lipid composition of Mel501 cells cultured in buffered (Mel501) and in acidic conditions (Mel501ac). Glycerophospholipid (GPL) and sphingolipid (SL) classes were identified and quantified by LC-MS/MS; Total cholesterol was quantified by GC-MS. (A) Amounts of GPL and SL relative to protein content (pmol of lipid/µg of proteins). (B) Amounts of cholesterol relative to protein content (pmol of lipid/µg of proteins. (C) The amount of GPL and SL subclasses expressed as pmol of lipid/µg of proteins. Mean values ± SD are shown (*n* = 6, Mel501; *n* = 4, Mel501ac) (*p < 0.05, Mel501 vs Mel501ac).

We determined the lipid profile of Mel501 cells cultured in buffered (Mel501) and in acidic conditions (Mel501ac) by LC-MS/MS. Data comparison highlighted numerous differences. Mel501ac had a higher content of glycerophospholipids (GPLs) (Figure 2(A)) and total cholesterol (Figure 2(B)), whereas the content of sphingolipids (SL) was comparable (Figure 2(A)). Among GPLs, the most abundant lipid subclasses were phosphatidylcholine (PC), followed by phosphatidylethanolamine (PE), phosphatidylglycerol (PG), PI and phosphadic acid (PA) (Figure 2(C)). As for SLs, we observed an abundant content of sphingomyelin (SM) and sulfatides (Sulf) (Figure 2(C)). The comparison of Mel501 and Mel501ac lipid subclass composition revealed differences. Specifically, in Mel501ac, we observed for GPLs an increased level of PCaa (PC carrying different fatty acids bound to the glycerol moiety by two ester linkages at both *sn*-1 and *sn*-2 position: di-acyl form, therefore aa means acyl-acyl), PI, all lyso-phospholipids (lyso-PLs) and we detected for SL a higher content of SM(OH) and lactosyl-ceramide (LacCer) (Figure 2(C)). These results provide evidence that Mel501 cells adaptation to growth in acidic conditions is associated with a higher content of specific GPL and SL subclasses.

### Analysis of molecular species of glycerophospholipid subclasses of Mel501 cells cultured in buffered (Mel501) and in acidic conditions (Mel501ac)

The membrane properties are not only affected by the amount and type of phospholipid polar heads, which can be argued by analysis of phospholipid classes content, but they are also influenced by fatty acids chain length and saturation. Therefore, we carried out further comparative analysis of individual lipid species between Mel501 and Mel501ac to address changes associated with cell growth adaptation to acidic microenvironment.

**Figure 3.**
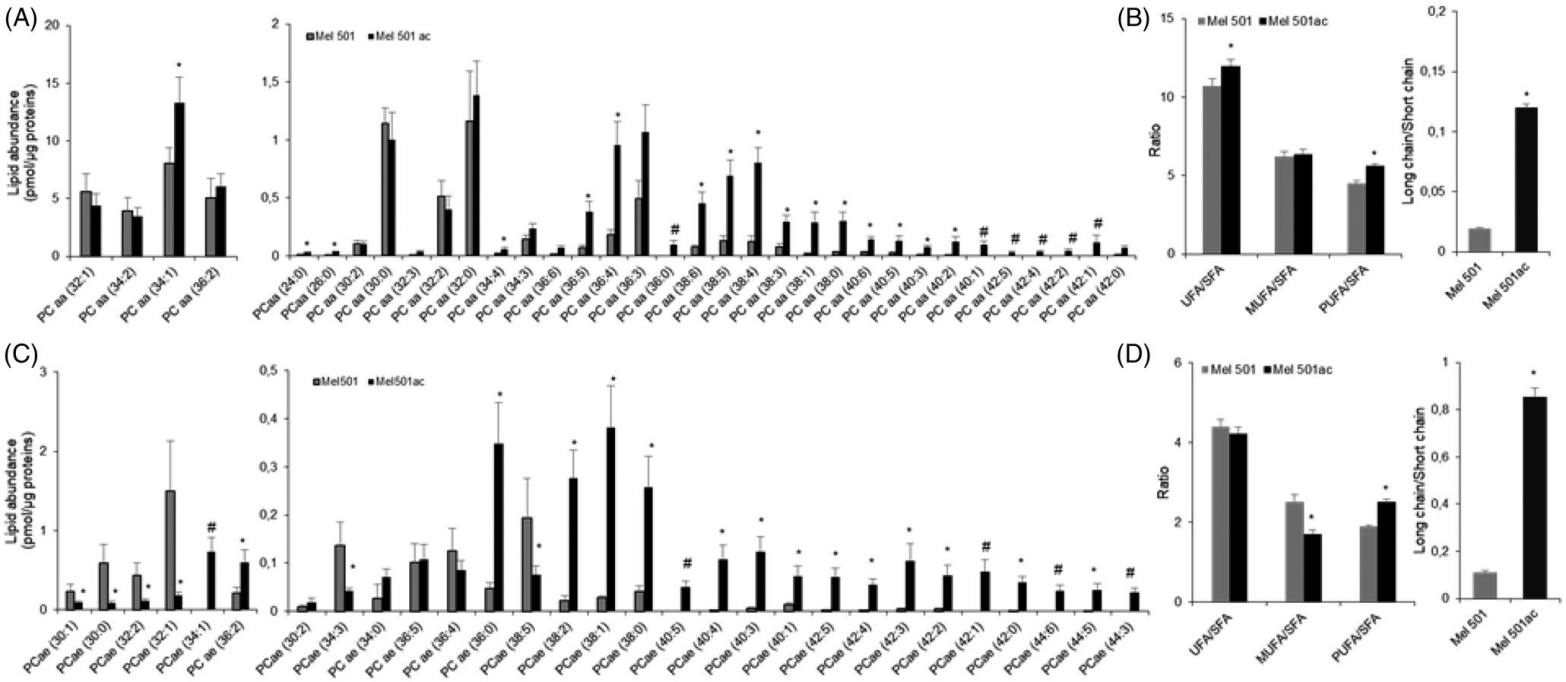
Molecular species of PCaa (A, B) and PCae (C, D) detected in Mel501 cells cultured in buffered (Mel501) and in acidic conditions (Mel501ac). Panels A and C show PCaa and PCae detected species, respectively. Data are expressed as pmol of lipid species/μg of proteins. Panels B and D show fatty acids saturation level and length in PCaa and PCae, respectively. Saturation level data are expressed as ratio of unsaturated to saturated PC species. Fatty acid length data are expressed as ratio of the total PC species with acyl chains whose sum was > 38 carbons (“long chains”) to PC molecules with acyl chains whose sum was < 36 carbons (“short chain”). Mean values ± SD (*n* = 6, Mel501; *n* = 4, Mel501ac) are shown (*p < 0.05, Mel501 vs Mel501ac; # lipid species exclusively found in Mel501ac). SFA, molecular species containing only saturated fatty acids; UFA: molecular species containing unsaturated fatty acids; MUFA: molecular species containing one double bond; PUFA: molecular species containing more than one double bond.

Analysis of PCaa and PCae (PC with a fatty acid at the *sn*-1 position linked by a vinyl ether linkage while the fatty acid at the *sn*-2 position linked by an ester linkage to the glycerol moiety: alkyl-acyl form, therefore ae means acyl-alkyl; these molecules are also known as plasmalogens) revealed 34 and 29 molecular species respectively, but 11 of them (6 of PCaa and 5 of PCae) were only detected in Mel501ac (Figure 3(A,C)). These Mel501ac specific species had long fatty acid chains (10 out of 11 with acyl chains whose sum was equal or higher than 38) and were mostly mono (4 species) or polyunsaturated (6 species).

Further, PCaa analysis showed that 17 lipids detected in both samples were increased in Mel501ac and none decreased (Figure 3(A)). To understand whether the degree of saturation and length of acyl chains was affected by extracellular acidic pH, we calculated the ratio of unsaturated (UFA) versus saturated (SFA) fatty acids in PCaa species. This revealed a significantly higher level of unsaturated species in Mel501ac (Figure 3(B)). When unsaturated PCaa lipids were further subdivided in species containing only one double bond, i.e. monounsaturated fatty acids (MUFA) or more than one double bond, i.e. polyunsaturated fatty acids (PUFA), it became evident that the increase of unsaturated species was due to an increase of polyunsaturated PCaa (Figure 3(B)). Regarding chain length, the 16 PCaa species with longer chains were either increased or only detected in Mel501ac (Figure 3(A)). Indeed, when we analysed the ratio of PCaa molecules with acyl chains whose sum was > 38 carbons (“long chains”) vs PCaa molecules with acyl chains whose sum was <36 carbons (“short chains”), we observed a significant increase for Mel501ac cells as compared to Mel501 (Figure 3(B)).

The presence of PCae was about 10% of total detected PC. As for PCae, a clear rearrangement of fatty acid chains could be observed, as 6 species were decreased and 16 were either increased or only detected in Mel501ac (Figure 3(C)). Although the ratio of unsaturated versus saturated species was comparable, a lower content of monounsaturated PCae and a higher content of polyunsaturated PCae was observed in Mel501ac (Figure 3(D). PCae species containing longer fatty acids were either increased or only detected in Mel501ac (Figure 3(C)) and a significant increase of species containing 38 or more carbons vs species containing 36 or less carbons was also observed (Figure 3(D)).

**Figure 4.**
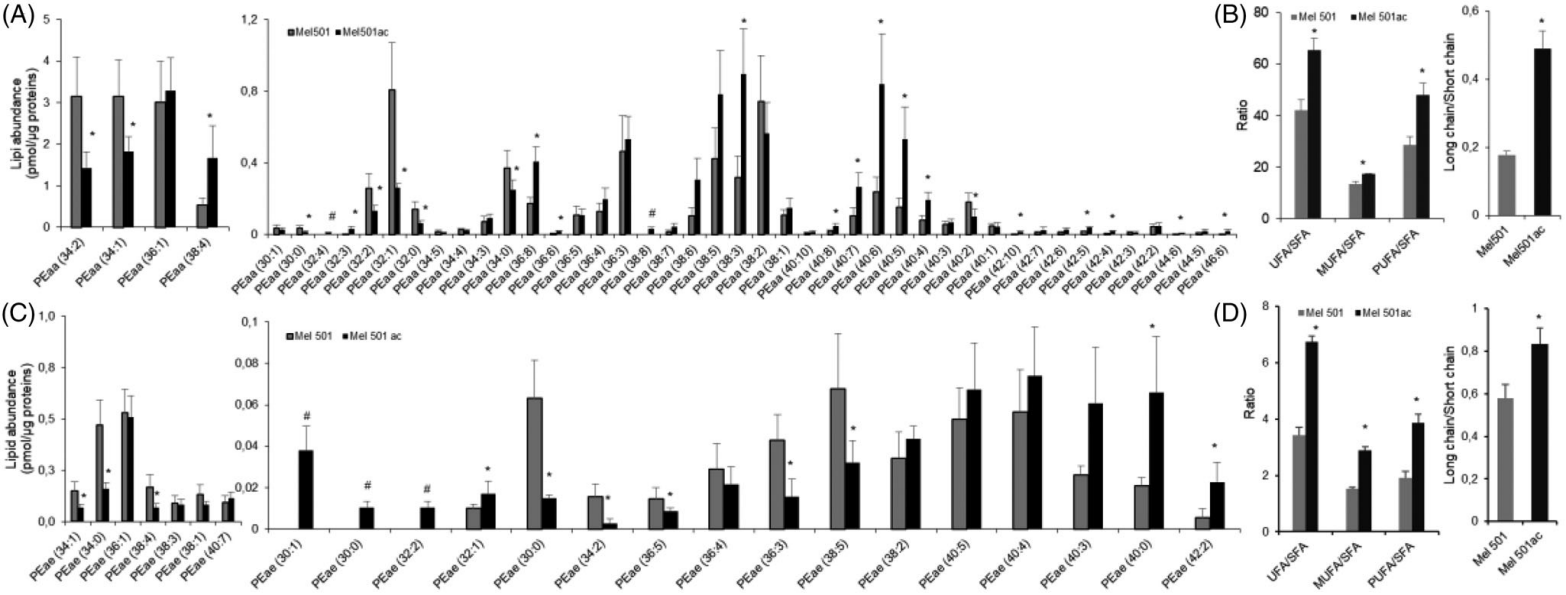
Molecular species of PEaa (A, B) and PEae (C, D) detected in Mel501 cells cultured in buffered (Mel501) and in acidic conditions (Mel501ac). Panels A and C show PEaa and PEae detected species, respectively. Data are expressed as pmol of lipid species/μg of proteins. Panels B and D show fatty acids saturation level and length in PEaa and PEae, respectively. Saturation level data are expressed as ratio of unsaturated to saturated PE species. Fatty acid length data are expressed as ratio of the total PE species with acyl chains whose sum was > 38 carbons (“long chains”) to PE molecules with acyl chains whose sum was < 36 carbons (“short chain”). Mean values ± SD (*n* = 6, Mel501; *n* = 4, Mel501ac) are shown (*p < 0.05, Mel501 vs Mel501ac; # lipid species exclusively found in Mel501ac). SFA, molecular species containing only saturated fatty acids; UFA: molecular species containing unsaturated fatty acids; MUFA: molecular species containing one double bond; PUFA: molecular species containing more than one double bond.

Analysis of the molecular species of PEaa and PEae detected 48 and 25 molecular species respectively, but 5 of them (2 PEaa and 3 PEae) were present only in Mel501ac. As shown in Figure 4, a clear rearrangement in favour of longer chains with more unsaturated bonds was observed in Mel501ac. For PEaa, the analysis showed that 16 molecular species were either increased or only detected in Mel501ac (Figure 4(A)). The ratio of unsaturated versus saturated PEaa species showed a higher unsaturation level due to an increase of both monounsaturated and polyunsaturated PEaa (Figure 4(B)). This was accompanied by a higher content of species containing longer acyl chains (Figure 4(B)). The content of PEae was about 9% of total detected PE and the rearrangement of carbon chains appeared more complex, as the three species only detected in Mel501ac were those with the shorter acyl chains (30:1; 30:0; 32:2) (Figure 4(C)). Nevertheless, like PEaa, the remodelling led to an increased ratio of unsaturated vs saturated fatty acids containing species (Figure 4D)) and to an increased ratio of longer vs shorter chains (Figure 4(D)).

The analysis of PI detected 13 species in both samples, with 7 of them increased in Mel501ac (Supplemental Figure 1). These species displayed a rearrangement of acyl chains consistent with that observed for PC and PE. The analysis of PG, phosphatidylserine (PS), and PA molecular species showed no significant changes between the two samples (data not shown). Altogether these results revealed a general trend of acyl chains lengthening and unsaturation in the most abundant membranes GPLs. In addition, it showed that the level of many individual lipid species significantly changed between Mel501 and Mel501ac (53 species increased, 22 decreased). Noteworthy, it also provides evidence that several PC and PE species (16 in total) were exclusively detected in Mel501ac. These results demonstrated that acidic extracellular pH induces clear changes in GPLs composition.

### Molecular species of lysophospholipids subclasses of Mel501 cells cultured in buffered (Mel501) and in acidic conditions (Mel501ac)

**Figure 5.**
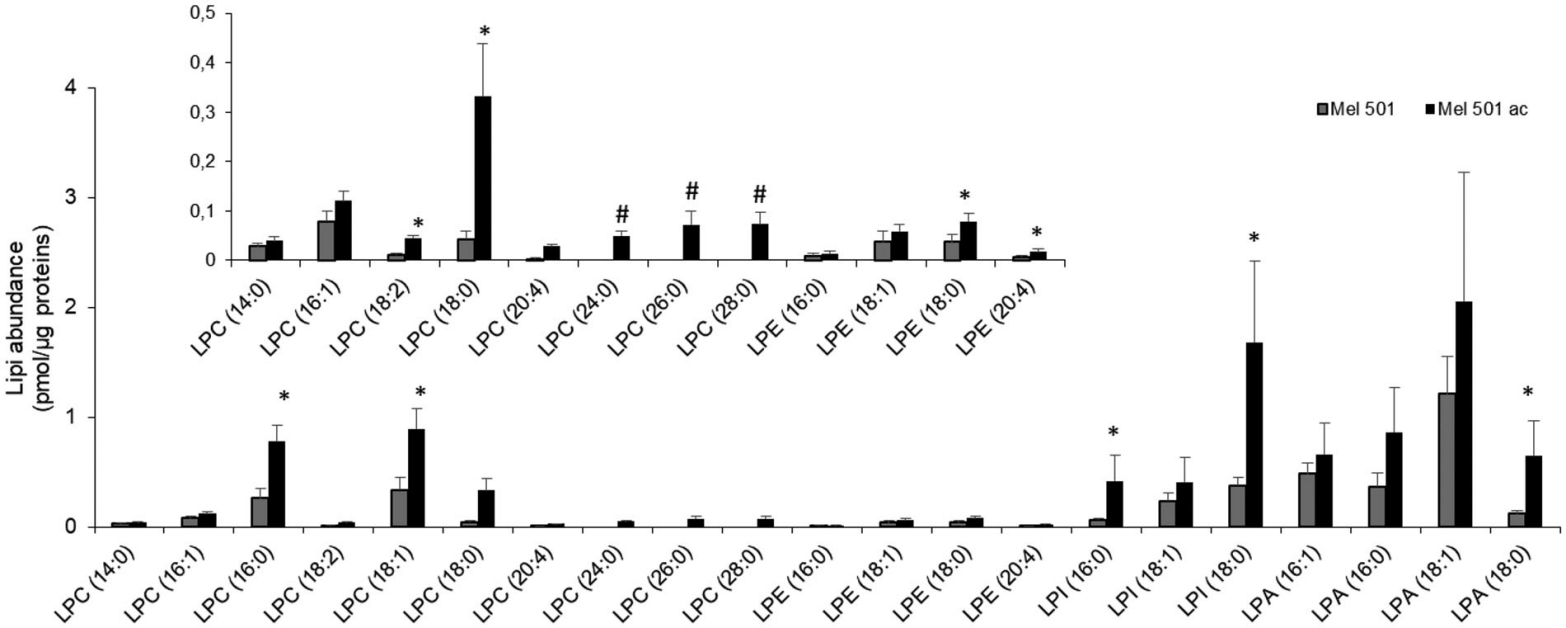
Molecular species of lysoPL detected in Mel501 cells cultured in buffered (Mel501) and in acidic conditions (Mel501ac). Lipid extracts were analysed by LC/MS-MS. Data are expressed as pmol of lipid species/μg of proteins. Mean values ± SD (*n* = 6, Mel501; *n* = 4, Mel501ac) are shown (*p < 0.05, Mel501 vs Mel501ac; # lipid species exclusively found in Mel501ac). The inserted panel expand the vertical axis to allow comparison of low abundance lipid subclasses.

Lyso-PLs are important both for the mechanical properties of biological membranes and as metabolites for intercellular signaling^30^^,^^31^. Our analysis detected 4 lyso-PL subclasses whose content was significantly increased in Mel501ac (Figure 2). Analysis of the individual molecular species showed 10 LPC species, with 3 of them only present in Mel501ac. These had very long saturated acyl chains (24:0, 26:0; 28:0) (Figure 5). Among LPC species that were common between the two samples, 5 were increased in Mel 501ac (Figure 5). Besides, 2 out of 3 LPI, 1 out of 4 LPA and 2 out of 4 LPE species were also significantly increased in Mel 501ac. This result clearly indicates that the increase of lyso-PL, independently of polar head group, is a hallmark of melanoma cell adaptation to low extracellular pH.

### Molecular species of sphingolipid subclasses of Mel501 cells cultured in buffered (Mel501) and in acidic conditions (Mel501ac)

**Figure 6.**
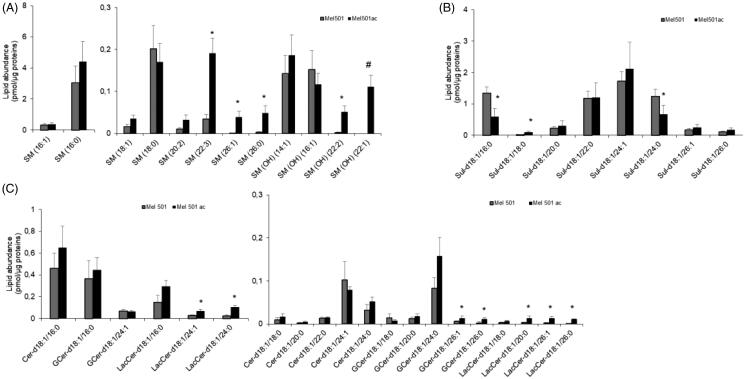
Molecular species of sphingolipid subclasses of Mel501 cells cultured in buffered (Mel501) and in acidic conditions (Mel501ac). Lipid extracts were analysed by LC/MS-MS. The amounts of lipid species of SL subclasses were expressed as pmol of lipid species/μg of proteins. (A) SM and SM(OH); (B) Sulfatides; (C) Cer, GCer and LacCer. Mean values ± SD (*n* = 6, Mel501; *n* = 4, Mel501ac) are shown (*p < 0.05, Mel501 vs Mel501ac; # lipid species exclusively found in Mel501ac). Cer: ceramide; GCer: glucosylceramide; LacCer: LactosylCeramide).

The analysis of SL individual species provided results consistent with those of GPLs. For SM and SM(OH), 3 out 8 and 2 out of 4 species were increased in Mel501ac, respectively. In addition, SM(OH) 22:1 was exclusively detected in Mel501ac. These species had the longest acyl chains among those detected (Figure 6(A)). The analysis also showed the presence of intermediates in the synthesis of SL (6 Cer species) and glycoSL (7 GCer and 6 LacCer species): 2 out of 6 GCer species and 5 out 6 LacCer species were increased in Mel501ac and were those with the longest acyl chains (Figure 6(C)). We also detected a remarkable amount of Sulf (8 species), but changes in their level (2 decreased, 1 increased) were not associated with acyl chain remodelling (Figure 6(B)). This set of results showed that acidic extracellular pH induces a rearrangement not only in GPLs but also of SM and SM(OH), in favour of longer carbon chains. Sulf, which possess a net negative charge, remain relatively unchanged in this condition.

### Analysis of total and free fatty acids of Mel501 cells cultured in buffered (Mel501) and in acidic conditions (Mel501ac)

**Figure 7.**
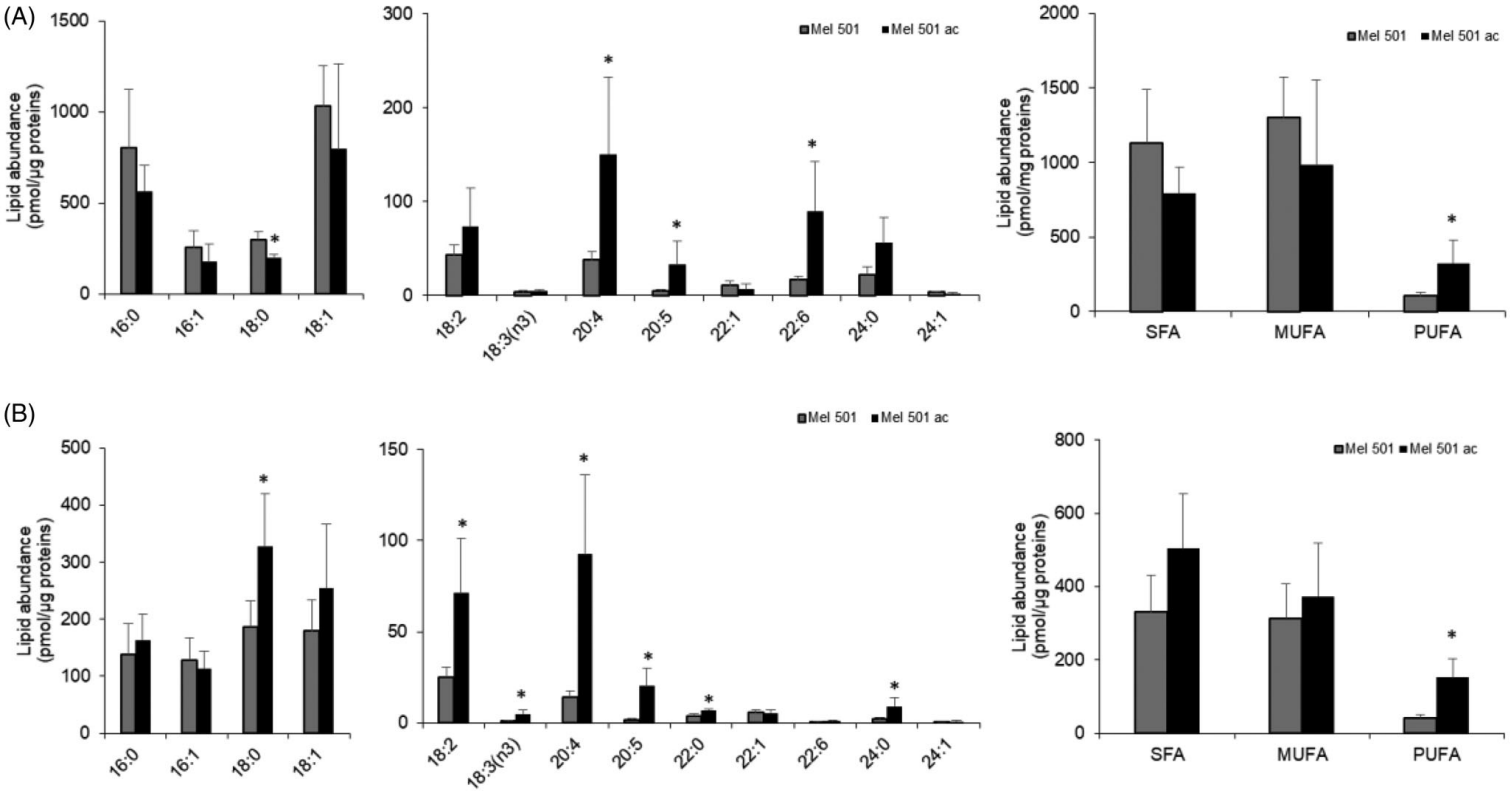
Fatty acid profiles of Mel501 cells cultured in buffered (Mel501) and in acidic conditions (Mel501ac). Cellular lipids were extracted and total (A) and free (B) fatty acids composition were analysed by LC-MS. Data are expressed as pmol of FA/µg of protein and are presented as mean + S.D (*n* = 6, Mel501; *n* = 4, Mel501ac) (*p < 0.05, Mel501 vs Mel501ac). SFA: saturated fatty acids; MUFA: mono-unsaturated fatty acids; PUFA: poly-unsaturated fatty acids.

To confirm the indication of a rearrangement of phospholipid carbon chains, we analysed both total and free fatty acids by LC-MS. As shown in Figure 7(A), total fatty acids composition displayed a significant increase of three PUFA species, i.e. arachidonic acid 20:4, eicosapentaenoic acid 20:5, docosahexaenoic acid 22:6, and the decrease of stearic acid 18:0. This evidence confirmed an increase of polyunsaturation in Mel501ac and, specifically, of PUFA species that are relevant as lipid mediator precursors. When free fatty acids were analysed, we observed an increase of three saturated species (18:0, 22:0, 24:0) of 4 polyunsaturated species, i.e. 18:2, 18:3, 20:4 and 20:5 (Figure 7(B)). The significant increase of 7 out of 13 detected free fatty acids agrees with the significant increased level of lysoPLs observed in Figure 7 and suggested a possible activation of phospholipase A2.

### Gene expression analysis of enzymes involved in fatty acids desaturation, elongation, and phospholipid remodelling

**Figure 8.**
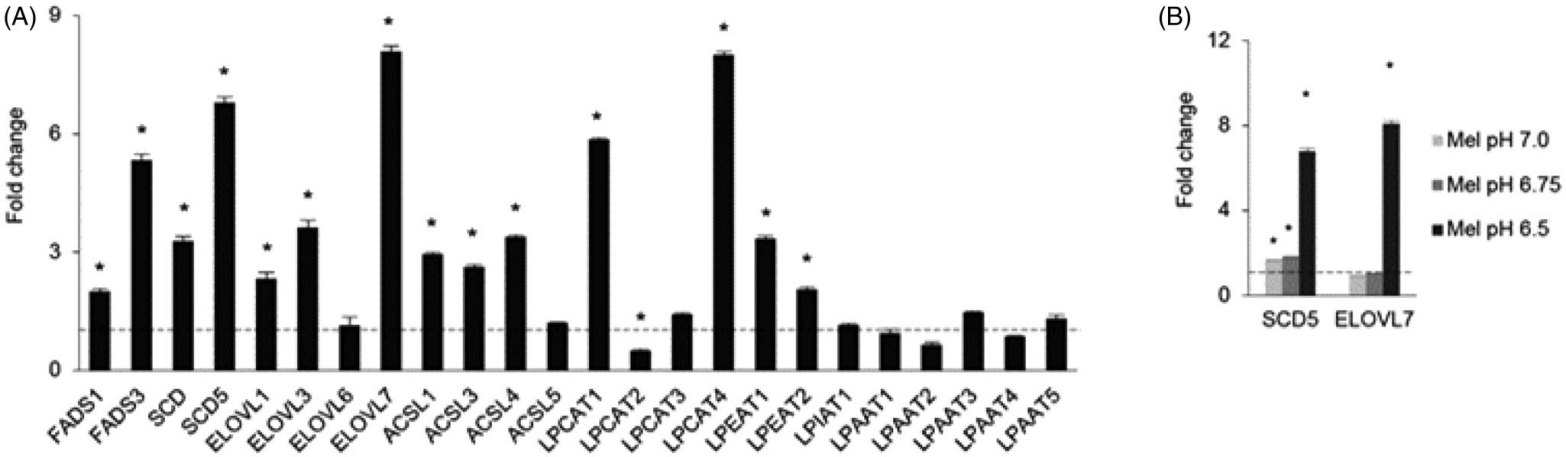
Gene expression analysis of Mel501 cells cultured in buffered (Mel501) and in acidic conditions (Mel501ac). Transcripts were determined in a StepOne RT-PCR machine (Applied Biosystems, USA) using SYBR^®^ Select Master Mix (Life Technologies). Panel (A) displays the fold change in Mel501ac with respect to Mel501. GAPDH gene was used as endogenous control (set 1). Panel (B) reports the fold change of SCD5 and ELOVL7 gene in Mel501 cultivated at progressively lower pH. For each experiment, the analysis was repeated three times in triplicate. The mean ± SD of a representative experiment is reported (*p < 0.05).

To understand whether the remodelling of phospholipids towards longer and more unsaturated acyl chains may be related to the activation of a specific transcriptional programme or due to the elimination of specific lipids, possibly by release of extracellular vesicles, we carried out qRT-PCR in Mel501ac vs Mel501 cells and determined the expression of genes involved in fatty acids desaturation, palmitate elongation, conjugation of long acyl chain fatty acids to acyl-CoA and lysosphospholipid acyl transferases involved in phospholipid fatty acids remodelling (Supplemental Table 1). Among genes expressed in Mel501 cells, results clearly demonstrated a specific upregulation of all desaturases, elongases and long-chain acyl-CoA synthases detected, except for ELOVL6 and ACLS5. In the case of lysosphospholipid acyl transferases, the adaptation to extracellular acidification induced up-regulation of specific enzymes, i.e. LPCAT1, LPCAT4, LPEAT1 and LPEAT2, whereas other members of the same family appeared down-regulated, i.e. LPCAT2, or remain unchanged, indicating that the higher expression of specific enzymes was required to survive extracellular low pH (Figure 8(A)). We also tested if the SCD5 desaturase and ELOVL7 elongase transcripts, which were showed the higher level of differential expression in Mel 501ac, were up-regulated in a pH dependent manner (Figure 8(B)). Results provided evidence that SCD5 up-regulation was an early event, detectable also when cells were cultivated at pH 6.75 and 7.0, whereas ELOVL7 transcript was up-regulated only at pH 6.5.

**Figure 9.**
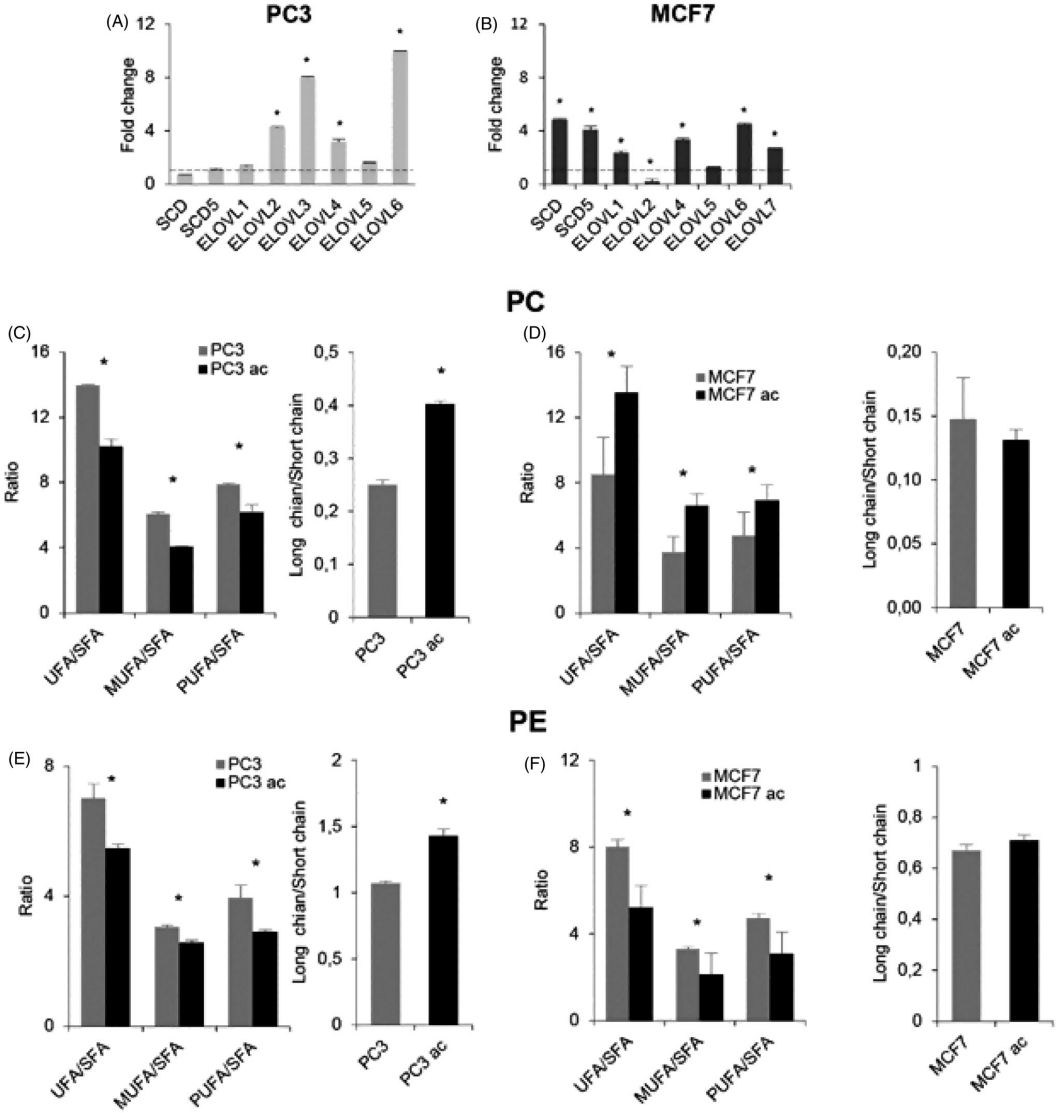
Analysis of PC3 and MCF7 cells cultured in buffered and acidic conditions. (A) and (B) show gene expression by qRT-PCR of the expressed deasuturase and elongase transcripts in PC3 and MCF7 cells cultivated at pH 6.5, respectively. Panels show the fold change. For each experiment, the analysis was repeated three times in triplicate. The mean ± SD of a representative experiment is reported (*p < 0.05). (C) and (D) show length and unsaturation of PC acyl chains in PC3 and MCF7 cells cultivated at pH 6.5, respectively. (E) and (F) show length and unsaturation of PE acyl chains in PC3 and MCF7 cells cultivated at pH 6.5, respectively. Saturation level data are expressed as ratio of unsaturated to saturated PC or PE species. Fatty acid length data are expressed as ratio of the total PC or PE species with acyl chains whose sum was > 38 carbons (“long chains”) to PC or PE molecules with acyl chains whose sum was < 36 carbons (“short chain”). Mean values ± SD (*n* = 6, PC3 or MCF7; *n* = 6, PC3ac or MCF7ac) are shown (*p < 0.05). SFA, molecular species containing only saturated fatty acids; UFA, molecular species containing unsaturated fatty acids; MUFA, molecular species containing one double bond; PUFA, molecular species containing more than one double bond.

Then, we analysed whether the transcriptional up-regulation of desaturases and elongases could be found in other cell lines cultured at acidic pH. We similarly adapted MCF7 breast cancer cells and PC3 prostate cancer cells to grow at pH 6.5 (Supplementary Figure S2), then tested the expression of a few desaturases (SCD and SCD5) and elongases (ELOVL1 to ELOVL7) (Figure 9(A,B)). Elongases expression profile was different as compared to Mel501 (as for example, PC3 and MCF7 express ELOVL2, which is not expressed by Mel501), but results showed that PC3 did not display higher desaturases level, but expressed higher levels of 4 out of 6 elongases (Figure 8(C)), whereas in MCF7 both desaturases and elongases (4 out of 6) were significantly up-regulated (Figure 8(D)). Analysis of PC and PE fatty chain saturation and length in MCF7 and PC3 adapted at low pH confirmed that, in correlation with a higher level of elongases but not desaturases expression in PC3, we detected a higher level of longer PC and PE fatty acid chains (Figure 9(C,E)) in cells adapted at low pH, whereas in correlation with a higher level of desaturases observed in MCF7, we observed a higher level of PC unsaturated species in cells adapted at low pH (Figure 9(D)), although fatty chain length was not significantly different.

## Discussion

Low extracellular pH is considered a key feature of tumour microenvironment^12^. The survival of tumour cells in this condition requires an extensive metabolic adaptation, including the structural changes of membrane phospholipids in order to fit their biophysical and biochemical properties to the new environment and support accelerated cell division and migration. To gain insight this process, we focussed our investigation on Mel501 melanoma cells adapted to survive at low pH (6.5) and compared their lipid signature with that of the same cells grown at physiological pH (7.4). As expected^21^^,^^22^, Mel501 cells change their morphology and acquire a behaviour consistent with a higher invasive potential when grown at low pH values, thus indicating that our model is suitable to investigate the molecular signatures associated to the growth in a hostile low pH microenvironment.

The analysis of the lipid content showed that Mel 501 cultured in acidic condition contains higher level of phospholipids with acyl chains whose sum was longer than 38 carbons and higher levels of polyunsaturated species. The diversity of fatty acids in membrane phospholipids occurs through two different pathways: the *de novo* pathway (Kennedy pathway) and the remodelling pathway (Lands’ cycle)^32^. The present findings indicate that low pH condition leads to changes in the remodelling of phospholipid fatty acids towards longer and more unsaturated carbon chains, with the presence of highly unsaturated species with 7–10 double bonds suggesting that both sn-1 and sn-2 carbon chains could be unsaturated. This was confirmed by the analysis of total fatty acids and was related to an up-regulation of desaturases and elongases expression, as demonstrated by qRT-PCR. Besides, analysis of desaturases and elongase expression in PC3 prostate cancer cells and MCF7 breast cancer cells further confirmed that high transcripts level of enzymes involved in fatty acid desaturation and elongation is a key feature of cancer cells cultivated in acidic microenvironment. In fact, consistently with higher expression of elongases but not desaturases in PC3 prostate cancer cells, PC and PE analysis showed that longer PC and PE species are increased in PC3 cells adapted at low pH. Instead, in MCF7 cells adapted at low pH, coherently with a higher expression of desaturases, unsaturated PC species are increased, whereas the fatty acid chain length is not significantly affected. Interestingly, in this case there is both significant up and down-regulation of elongase transcripts.

Mel501 analysis also showed higher levels of polyunsaturated free fatty acids and coherently, an increase of lysophospholipid species. This finding may be explained by an increased activity of phospholipases. The presence of acyl chains longer than 18 carbons in LPC and LPE, which were not present in LPA, and the up-regulation of specific LPC acyltransferases but not of LPA acyltransferases transcripts indicated that these species could not derive from the *de novo* synthesis but from phospholipid remodelling routes. The increase of PI is consistent with previous studies on melanoma cells lipidome in stress conditions, i.e. temperature increase and UVA exposure^18^^,^^19^. More relevant, the increase of PI has been also recently described to be a potential biomarker of melanoma cell lines with metastatic potential. However, in this case the increase was due to saturated and monounsatured PI species^20^, whereas we observed a higher increase of species containing polyunsaturated acyl chains. The higher level of PI is also relevant because this phospholipid is key determinant of net membrane surface charge and its level can affect the functional interactions with positively charged regions of membrane proteins, modulating their localisation and activity.

The high presence of PUFA could affect membrane fluidity^33^^,^^34^, which in turn affects many cellular functions that are dependent on membrane dynamics. However, in terms of fluidity differences between mono and polyunsaturated phospholipids are limited as compared to the differences between saturated and unsaturated phospholipids, so the role of polyunsaturation versus monounsaturation must be further elucidated. Incorporation of polyunsaturated fatty acids also facilitates membrane flexibility and deformation, promoting membrane fusion and/or fission^35^^,^^36^. Consistent with this finding, it has been previously demonstrated that melanoma cells cultured at acidic pH release a higher number of exosomes^37^. In fact, these vesicles originate from the invagination of late endosomes and accumulate into multivesicular bodies and their biosynthesis is strongly dependent of fission/fusion events promoted by the ESCRT complex. The presence of polyunsaturated species also influences lipid bilayer thickness and introduces lipid packing defects^33^^,^^34^. Since cholesterol interferes with acyl-chain packing and reduce the flexibility of unsaturated acyl chains, its higher content in Mel501ac could allow cells to minimise the increased movement across their membranes, that could be associated with a higher level of unsaturation. The increase in acyl chains length may be associated with a promotion of membrane thickness counteracting the tendency of membranes rich of polyunsaturated phospholipids to be thinner than their saturated counterparts. The high level of PUFA in Mel 501ac is important for the biosynthesis of lipid messengers. Polyunsaturated fatty acids such as arachidonic acid, docosahexaenoic acid, and eicosapentaenoic acid can be converted into biologically active eicosanoids^38^, that can be active both in the onset of inflammation and in its resolution.

As for SL, a significant alteration in their metabolism was the increase of LacCer. LacCer is not only a metabolic intermediate in the synthesis of glycoSL, generated by the action of LacCer synthase on GCer or by the degradation of complex glycoSL, but it is also a bioactive lipid with a specific role in inflammation^39^, further suggesting that the alteration of sphingolipid metabolism induced by low extracellular pH may be also involved in the modulation of the inflammation related pathways.

The role of pH on the structure and dynamics of membrane lipids has been investigated on membrane model systems. Studies reported that low pH enhances membrane thickness, influences spontaneous curvature and induces head group protonation^40^^,^^41^. However, when cells are adapted to grow at low pH, only the external leaflet must adapt physically to an elevated H^+^ concentration, whereas the rest of the cell membranes must adapt to support this growth condition without being directly exposed to low pH. ER and plasma membrane show differences in their lipid composition, as sterols and sphingolipids are present at a low percentage in the ER and their abundance increase in the route to the Golgi and the plasma membrane^42^. Similarly, phospholipids are more saturated at the plasma membrane than at the ER. For this reason, further insight on the phospholipid composition of specific cell fractions is needed to understand the role of phospholipid remodelling towards longer and unsaturated acyl chains in cells exposed to a low extracellular pH, as the function of specific subcellular organelle biomembranes could be affected.

## Conclusions

This study provides an exhaustive analysis of changes induced by microenvironmental acidosis in the lipid composition of malignant human cells. Overall findings support the evidence that acidic condition does not simply induce a modification of tumour cell lipid content, rather the structural lipid composition looks deranged as compared to that of normal cells. Analysis of the individual lipid species may represent a source for further investigation aimed at unravelling potential cancer biomarker related to the invasive capacity of the tumour. The peculiar lipid signature impressed by extracellular acidification is associated with a specific transcriptional programme not only in Mel501 cells but also in breast and prostate cancer cell lines MCF7 and PC3, implicating that expression changes of desaturases and elongases, are not limited to melanoma cells but are a widespread event in other cancer cells, important for cell survival^43^. In this context, our results provide further clues to the current use of phospholipid and fatty acid metabolism inhibitors as potential targets for anti-cancer therapies.

## Supplementary Material

Supplemental Material
