# Supplementary material for: Lipidomic analysis of cancer cells cultivated at acidic pH reveals phospholipid fatty acids remodelling associated with transcriptional reprogramming
Source: J Enzyme Inhib Med Chem. 2020 Apr 20;35(1):963–73. doi: 10.1080/14756366.2020.1748025 (PMC7191909; doi:10.1080/14756366.2020.1748025)
Supplement: Supplemental Material [file IENZ_A_1748025_SM6072.pdf]

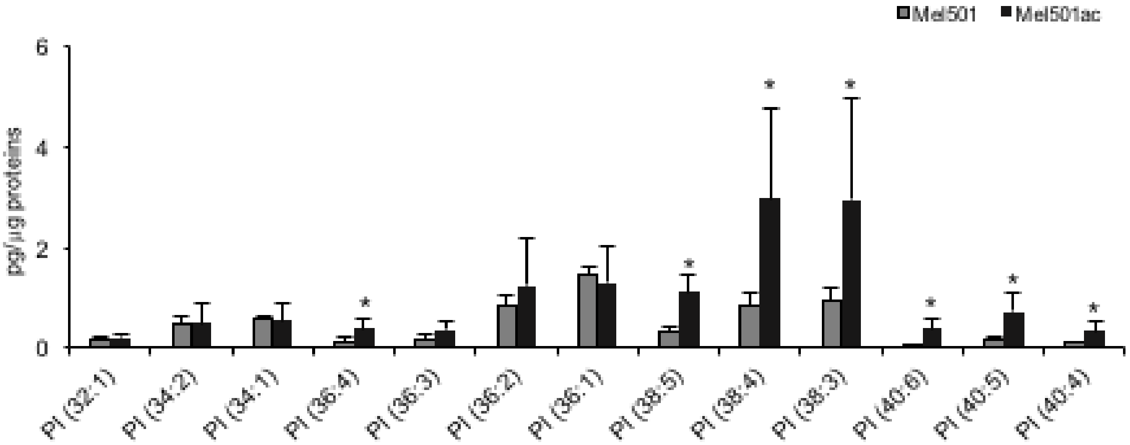

Figure S1. Molecular species of PI of Mel501 cells cultured in buffered (Mel501) and in acidic conditions (Mel501ac). Lipid extracts were analysed by LC/MS-MS. Data are expressed as pg of lipid species/µg of proteins. Mean values ±S.D. (n = 6, Mel501; n=4, Mel501ac) are shown (*p<0.05, Mel501 vs Mel501ac; # lipid species exclusively found in Mel501ac). The inserted panels expand the vertical axis to allow comparison of low abundance lipid subclasses.

	Symbol	Entry	Forward	Reverse
**Palmitate elongation**				
	**ELOVL1**	NM_001256402.1	TGAAGCACGCAGTTCCTGAT	GGAGAAGAGGAAGAGCCAGG
	**ELOVL2**	NM_017770.3	CGCTGCGGATCATGGAACAT	GTCCAACATGAACCACCCTCT
	**ELOVL3**	NM_152310.2	ACCTCATTCCCCATAGCCCT	TGCCCCCAGGATACTGAAGA
	**ELOVL4**	NM_022726.3	AGTTCTACCGCTGGACCTG	TGATCCCATGAATAACTCTCTGAA
	**ELOVL5**	NM_021814.4	GCGCTTGATTCATCCTTCGG	CCATTTGAAAACCTTTTAGCCCA
	**ELOVL6**	NM_001130721.1	AAAGCACCCGAACTAGGAGA	GCCCGCAAGGCATAGTAAGA
	**ELOVL7**	NM_024930.2	AGATGCTGATCCAAGAGTTGAAG	TGAGTTCAAAGGGCTTGCGA
**Desaturation**				
	**SCD**	NM_005063.4	TTCCCGACGTGGCTTTTTCT	AGCCAGGTTTGTAGTACCTCC
	**SCD5**	NM_001037582.2	ACTCTGCTCTGGGCCTACTT	GAGTACTTGTGGTGGGCTCG
	**FADS1**	NM_013402.4	CAGCTATGGCCCCCGAC	ACAAAGGGATCCGTGGCAT
	**FADS2**	NM_004265.3	CAGTCGGCAGGCAGCAT	GGAAGGCATCCGTTGCATCT
	**FADS3**	NM_021727.4	AGGACTCGTGCGTGCAG	GAAGGCATCCGTGGCGT
**Conjugation of long acyl chain fatty acids to acyl CoA**				
	**ACSL1**	NM_001286710.1	GAGCTTTTGCAGCACTCACC	ACAAGGGCCATTATTTGACACC
	**ACSL3**	NM_004457	ATTGTGCATACCATGGCTGCAGTG	TCTGGAATCCTTTCTGCCATCCCA
	**ACSL4**	NM_004458	TGGGCATTCCTCCAAGTAGACCAA	ACTGGCCTGTCATTCCAGCTATCA
	**ACSL5**	NM_016234	TCAGTCATCACATTCTTCCGGGCA	CCAGCTTCACGTAATTGCAAGCCA
	**ACSL6**	NM_015256	AGCTGGCCTGCTACACATATTCCA	TCCACATGCTCTAGCAGAAGCACA
**Lysophospholipid acetyl trasferases**				
	**LPCAT1**/ AGPAT9	NM_024830	GGCGGAACCCCTTCGTG	CACAACCTTCCTCCACAGGG
	**LPCAT2**/ AGPAT11	NM_017839	AGTATGTGATTGGCCTGGCTGTCT	TCCAAGGGAAGCCTGTAGAATGGT
	**LPCAT3**/ MBOAT5	NM_005768	AACAGACCATCCACTGGCTCTTCA	TCAGGAAGAAGATGTGGCCAAGGA
	**LPCAT4**/ MBOAT2	NM_001321266.1	TTTGCATGGACGCTAGCTGA	GTTGGACTGAAGGAGGTTCGT
	**LPEAT2**/ LPCAT4	NM_153613	GTAGGGAGCTTACCTGTGATTGT	CCACATAGCCAGCGGACA
	**LPEAT1**/ MBOAT1	NM_001080480.2	CAACCACCCAAGAGCACCC	ACCACAAAATTCACCTGGTCC
	**LPIAT1**/ MBOAT7	NM_024298.4	GTGGGAATCATGACAGGCCC	GGATCATGTAGAAGAGGCGGG
	**LPAAT1**/ AGPAT1	NM_006411.3	CCCACCATTCCTACCGCTAT	ATCCATTCTGGCCACCTCAG
	**LPAAT2**/ AGPAT2	NM_001012727	CCCGTGGGCCTCATCATGTA	CGATGGGCACGTTCTCCC
	**LPAAT3**/ AGPAT3	NM_001037553.1	TTGTCTTCGTGGTGAGTGGT	ATGACCAGTTGGCTCCAGAGT
	**LPAAT4**/ AGPAT4	NM_020133.2	ATGCACCATCTTCACGGACC	GACCTTGGAGCCCCCTAACA
	**LPAAT5**/ AGPAT5	NM_018361.3	TTGCTCAGCATGGAGGAATCT	TGCCTGACTAGCTGAAAGGAC

Table S1. Primers used for qRT-PCR. The symbols used in the main text are indicated in bold.
